# Clinical and Functional Outcomes of Posterior Endoscopic Decompression for Symptomatic Cervical Disc Herniation

**DOI:** 10.7759/cureus.110822

**Published:** 2026-06-14

**Authors:** Adam Mahendra Teja, Muhammed Anzar, Cheol Wung Park

**Affiliations:** 1 Department of Orthopedics, Apollo Jabalpur Hospitals, Jabalpur, IND; 2 Department of Spine Surgery, Malabar Medical College Hospital and Research Centre, Modakkallur, IND; 3 Department of Neurosurgery, Daejeon Woori Hospital, Daejeon, KOR

**Keywords:** cervical disc herniation, cervical radiculopathy, endoscopic decompression, minimally invasive spine surgery, neck disability index

## Abstract

Introduction: Posterior endoscopic decompression is a minimally invasive alternative for symptomatic cervical disc herniation management. We aimed to evaluate the clinical and functional outcomes of posterior endoscopic decompression in patients with symptomatic cervical disc herniation. The objectives were to assess postoperative improvement in pain and disability, determine patient-reported functional outcomes using the Modified MacNab criteria, and analyze the influence of demographic and clinical variables on surgical success.

Materials and methods: This single-center study included 145 patients with symptomatic cervical disc herniation treated with posterior endoscopic decompression. Preoperative and postoperative assessments included Visual Analog Scale (VAS) scores for arm and neck pain and Neck Disability Index (NDI) scores. Patients were followed up at 1, 6, and 12 months postoperatively. Functional outcomes were evaluated using the Modified MacNab criteria. Statistical analysis was performed using repeated-measures analysis of variance and Pearson’s chi-squared test, with significance set at p < 0.05.

Results: The study included 82 (56.6%) males and 63 (43.4%) females with a mean age of 48.3 ± 9.7 years. The most commonly affected levels were C5-C6 in 61 (42.1%) patients and C6-C7 in 59 (40.7%). Mean arm pain VAS score significantly improved from 7.6 ± 1.2 preoperatively to 1.9 ± 1.0 at 12 months (p < 0.001). Mean neck pain VAS score decreased from 5.1 ± 1.8 to 2.2 ± 1.1 (p < 0.001), while mean NDI score improved from 54.3 ± 10.5 to 19.8 ± 8.2 (p < 0.001). According to the Modified MacNab criteria, excellent outcomes were observed in 72 patients (49.7%), and good outcomes were observed in 48 patients (33.1%), resulting in an overall success rate of 82.8%. Paracentral disc herniation demonstrated significantly better outcomes than foraminal herniation (p = 0.042).

Conclusion: Posterior endoscopic decompression provides significant pain relief, functional improvement, and favorable clinical outcomes in patients with symptomatic cervical disc herniation, representing an effective and minimally invasive surgical treatment option.

## Introduction

Cervical disc herniation is a common degenerative spinal disorder and an important cause of cervical radiculopathy, frequently presenting with neck pain, radiating arm pain, sensory disturbances, and motor weakness [1]. Compression and inflammation of the exiting cervical nerve root secondary to posterolateral or foraminal disc protrusion can significantly impair daily activities and quality of life [2]. Conservative treatment modalities, including analgesics, physiotherapy, cervical traction, and epidural steroid injections, are generally considered first-line management strategies [3]. However, a considerable proportion of patients continue to experience persistent symptoms despite adequate nonsurgical treatment, which necessitates surgical intervention.

Anterior cervical discectomy and fusion have traditionally been regarded as standard surgical treatments for symptomatic cervical disc herniation. Although this technique provides satisfactory decompression and clinical outcomes, it is associated with several disadvantages, including adjacent segment degeneration, reduced cervical mobility, dysphagia, and implant-related complications [4,5]. In recent years, minimally invasive spinal procedures have gained increasing attention because of their ability to minimize soft tissue trauma, reduce postoperative pain, shorten hospital stays, and facilitate faster recovery while preserving spinal stability.

Posterior endoscopic decompression has emerged as a promising, minimally invasive alternative for the selection of patients with lateral cervical disc herniation. This technique enables direct visualization and decompression of the affected nerve root through a small posterior approach while preserving the intervertebral disc and motion segment [6,7]. Advancements in endoscopic optics, instrumentation, and irrigation systems have improved the safety and efficacy of the procedure. Nevertheless, the clinical effectiveness of posterior endoscopic decompression and the factors influencing surgical outcomes remain areas of ongoing investigation.

This study aimed to evaluate the clinical and functional outcomes of posterior endoscopic decompression in patients with symptomatic cervical disc herniation. Specifically, postoperative changes in arm and neck pain were assessed using the Visual Analog Scale (VAS), functional disability was evaluated using the Neck Disability Index (NDI), and overall patient-reported outcomes were determined using the Modified MacNab criteria. In addition, clinical outcomes were compared across selected demographic and disease-related subgroups.

## Materials and methods

Study design and ethical approval

This single-center study was conducted at Apollo Jabalpur Hospitals, Jabalpur, India, from March 2024 to December 2025. The study protocol was approved by the Ethical Committee of Apollo Jabalpur Hospitals (AJBPH/2024/520 dated 20.2.24), and all procedures were conducted in accordance with the ethical principles of the Declaration of Helsinki and the Strengthening the Reporting of Observational Studies in Epidemiology (STROBE) guidelines [8]. Written informed consent was obtained from all participants before enrolment and surgical intervention.

Sample size estimation

Sample size estimation was performed a priori using G*Power software version 3.1.9.7 (Heinrich Heine University, Düsseldorf, Germany). Based on the findings of a previously published study, an anticipated clinical success rate of 85% following posterior endoscopic decompression was considered [9]. Assuming a two-sided 95% confidence interval (CI) with a precision margin of ±6% and statistical power of 80%, the minimum required sample size for estimating a single proportion was calculated to be 132 patients. The final target sample size was increased to 145 patients to compensate for the anticipated loss to follow-up of 10%.

Study population

A total of 145 consecutive patients diagnosed with symptomatic cervical disc herniation were included in this study. Patients were screened according to predefined inclusion and exclusion criteria. Individuals presenting with unilateral cervical radiculopathy secondary to posterolateral or foraminal cervical disc herniation documented on preoperative imaging and who had failed at least six weeks of conservative management were considered eligible. Conservative therapy included physiotherapy, analgesics, cervical traction, and epidural steroid injection. Patients were required to have a VAS score of ≥4 for arm pain at baseline [10]. Patients with central cervical disc herniation associated with myelopathy, cervical instability, spondylolisthesis, ossification of the posterior longitudinal ligament, prior cervical surgery at the same level, active infection, or traumatic cervical injury were excluded from the study.

Preoperative assessment

All participants underwent detailed preoperative clinical, neurological, and radiological evaluation. Neurological examination included assessment of motor strength, sensory deficits, deep tendon reflexes, and signs of nerve root compression. Pain intensity was assessed using the VAS, a 10-cm scale ranging from 0 (no pain) to 10 (worst imaginable pain). Functional disability was evaluated using the NDI to assess disabilities related to cervical spine disorders. The NDI consists of 10 items assessing pain intensity, personal care, lifting, reading, headaches, concentration, work, driving, sleeping, and recreation, with each item scored from 0 to 5 [11]. The total score ranges from 0 to 50, with higher scores indicating greater functional disability. All patients underwent standard preoperative clinical and radiological evaluation. Magnetic resonance imaging (MRI) was used to identify the level and characteristics of cervical disc herniation. Additional diagnostic investigations were performed when clinically indicated.

Surgical procedure

All surgical procedures were performed under general anesthesia by a trained spinal surgeon with the patient positioned prone and the cervical spine maintained in slight flexion. Intraoperative localization of the affected spinal level was achieved using fluoroscopic guidance in anteroposterior and lateral projections. A paramedian skin incision measuring approximately 1.5-2 cm was made 1 cm lateral to the spinous process at the affected level. Sequential dilators were introduced to establish the working corridor, followed by insertion of a rigid endoscope with an outer diameter of 6.9 mm and a 4.3 mm working channel. The endoscopic system was connected to a high-definition camera and a continuous saline irrigation unit.

The medial portions of the inferior articular process and facet joints were identified using endoscopic visualization. Foraminotomy and flavectomy were subsequently performed using a high-speed diamond burr and Kerrison punch, respectively, to expose the lateral dura and the exiting nerve root. The herniated disc fragments were carefully mobilized and removed using endoscopic forceps under direct visualization. Aggressive intradiscal curettage was avoided to preserve disc height and segmental stability. Hemostasis was achieved using bipolar radiofrequency coagulation. After adequate neural decompression, the endoscope was withdrawn, and the incision was closed using a subcutaneous suture and skin adhesive.

Outcome measures

Patients were evaluated preoperatively and postoperatively at 1, 3, 6, and 12 months and annually thereafter. The primary outcome measures included arm pain intensity assessed using the VAS and functional disability assessed using the NDI. Secondary outcome measures included neck pain intensity measured using the VAS and overall functional recovery evaluated using the Modified MacNab criteria [12]. According to the Modified MacNab classification, outcomes were categorized as excellent, good, fair, or poor based on symptomatic improvement and return to normal activities (Table 1). Patients presenting with persistent or worsening symptoms during follow-up underwent further clinical and radiological evaluation as deemed necessary by the treating surgeon.

**Table 1:** Modified Macnab criteria for the assessment of clinical outcomes. Reproduced from Ozger and Kaplan [12] under the creative commons CC BY-NC-ND 4.0 license.

Outcome	Characteristics
Excellent	No pain; no restriction of mobility; return to normal work and level of activity
Good	Occasional nonradicular pain; relief of presenting symptoms; able to return to modified work
Fair	Some improved functional capacity; still handicapped and/or unemployed
Poor	Continued objective symptoms of root involvement; additional operative intervention needed at the index level, irrespective of the length of postoperative follow-up

Postoperative follow-up examinations were conducted in the outpatient department by an independent orthopedician, who was not involved in the surgical procedure. Patients who failed to attend scheduled visits were contacted telephonically to assess long-term clinical outcomes. The minimum follow-up period for inclusion in the final analysis was 12 months.

Statistical analysis

All statistical analyses were performed using IBM SPSS Statistics for Windows, Version 26 (Released 2019, IBM Corp., Armonk, New York). Continuous variables were expressed as mean ± standard deviation (SD), while categorical variables were presented as frequencies and percentages. Normality of data distribution was assessed using the Shapiro-Wilk test. Preoperative and postoperative longitudinal outcome measures, including VAS and NDI scores, were compared using repeated-measures analysis of variance with Greenhouse-Geisser correction. Categorical variables, including Modified MacNab outcomes and subgroup analyses, were evaluated using Pearson’s chi-square test or Fisher’s exact test, where appropriate. Statistical significance was defined as a two-sided p < 0.05.

## Results

All 145 enrolled patients completed the minimum 12-month follow-up and were included in the final analysis. No patients were lost to follow-up. Baseline demographic and clinical characteristics of the study population are shown in Table 1. A total of 145 patients were included in the study, including 82 males (56.6%) and 63 females (43.4%). The mean age of the participants was 48.3 ± 9.7 years. The most commonly affected cervical levels were C5-C6 in 61 patients (42.1%) and C6-C7 in 59 (40.7%). Paracentral disc herniation was observed in 88 patients (60.7%), and foraminal herniation was noted in 57 patients (39.3%). The mean preoperative VAS pain score was 7.6 ± 1.2, and the mean NDI score was 54.3 ± 10.5 (Table 2).

**Table 2:** Baseline demographic and clinical characteristics of the study participants (n = 145). Data are presented as mean ± standard deviation (SD), median (interquartile range as IQR), or frequency n (%). MRI: magnetic resonance imaging; VAS: Visual Analog Scale; NDI: Neck Disability Index.

Characteristic		Value
Age, mean ± SD	Years	48.3 ± 9.7
Sex, n (%)	Male	82 (56.6)
	Female	63 (43.4)
Body mass index, mean ± SD	kg/m²	26.1 ± 3.4
Symptom duration, median (IQR)	Months	6 (4-9)
Affected level, n (%)	C4-C5	12 (8.3)
	C5-C6	61 (42.1)
	C6-C7	59 (40.7)
	C7-T1	13 (9.0)
Herniation type (MRI), n (%)	Paracentral	88 (60.7)
	Foraminal	57 (39.3)
VAS arm pain, mean ± SD	Preoperative	7.6 ± 1.2
NDI, mean ± SD	Preoperative	54.3 ± 10.5

The postoperative clinical outcomes demonstrated significant improvements in pain and functional disability during the follow-up period (Table 3). The mean VAS arm pain score ranged from 7.6 ± 1.2 preoperatively to 1.9 ± 1.0 at 12 months (p < 0.001). Similarly, the mean neck pain VAS score reduced from 5.1 ± 1.8 to 2.2 ± 1.1 (p < 0.001). The mean NDI score also improved significantly from 54.3 ± 10.5 preoperatively to 19.8 ± 8.2 at final follow-up (p < 0.001).

**Table 3:** Longitudinal comparison of clinical outcome measures before and after posterior endoscopic decompression. Data are presented as mean ± SD. Statistical analysis was performed using repeated-measures analysis of variance with Greenhouse-Geisser correction. *Statistically significant at p < 0.05. VAS: Visual Analog Scale; NDI: Neck Disability Index.

Outcome		Preoperative (n = 145)	1 month (n = 145)	6 months (n = 142)	12 months (n = 138)	F value	p‑value
Arm pain (VAS, 0-10)	Mean ± SD	7.6 ± 1.2	3.1 ± 1.1	2.0 ± 1.0	1.9 ± 1.0	487.3	< 0.001*
Neck pain (VAS, 0-10)	Mean ± SD	5.1 ± 1.8	2.9 ± 1.2	2.3 ± 1.0	2.2 ± 1.1	128.6	< 0.001*
NDI (0-100)	Mean ± SD	54.3 ± 10.5	30.1 ± 8.9	20.9 ± 7.6	19.8 ± 8.2	310.4	< 0.001*

According to the Modified MacNab criteria, excellent outcomes were achieved in 72 patients (49.7%) and good outcomes were observed in 48 patients (33.1%) (Table 4). Fair and poor outcomes were noted in 16 (11.0%) and nine (6.2%) patients, respectively. The overall success rate, defined as the combination of excellent and good outcomes, was observed in 120 patients (82.8%).

**Table 4:** Distribution of Modified MacNab outcomes at final follow-up. Data are presented as frequency, n (%). Modified MacNab outcomes were assessed at final outpatient follow-up or telephone interviews. The overall success rate was calculated as the sum of excellent and good outcomes. The CI was calculated using the Wilson score method. CI: confidence interval.

MacNab grade	n (%)	CI at 95%
Excellent	72 (49.7%)	41.6-57.7%
Good	48 (33.1%)	26.0-41.1%
Fair	16 (11.0%)	6.9-17.2%
Poor	9 (6.2%)	3.3-11.4%
Success rate (Excellent + Good)	120 (82.8%)	75.9-88.3%

Subgroup analysis demonstrated higher success rates among patients with a symptom duration ≤6 months than among those with a longer symptom duration, although the difference was not statistically significant (p = 0.062) (Table 5). Patients with paracentral disc herniation had significantly better outcomes than those with foraminal herniation (p = 0.042). No statistically significant differences were observed in age, surgical level, or preoperative VAS scores.

**Table 5:** Subgroup analysis of Modified MacNab outcomes according to demographic and clinical variables. Data are presented as frequency n (%). Statistical analysis was performed using Pearson’s chi-square test. Fisher’s exact test was applied where expected cell counts were less than 5. Success was defined as excellent and good outcomes according to the Modified MacNab criteria. *Statistically significant at p < 0.05. VAS: Visual Analog Scale.

Subgroup		Excellent + Good n (%)	Fair + Poor n (%)	Test value	p-value
By symptom duration	≤6 months (n = 51)	46 (90.2%)	5 (9.8%)	χ² = 3.48	0.062
	>6 months (n = 94)	74 (78.7%)	20 (21.3%)		
By surgical level	C5-C6 (n = 62)	54 (87.1%)	8 (12.9%)	χ² = 2.18	0.336
	C6-C7 (n = 52)	42 (80.8%)	10 (19.2%)		
	Other levels (n = 31)	24 (77.4%)	7 (22.6%)		
By age group	Age < 45 years (n = 76)	66 (86.8%)	10 (13.2%)	χ² = 2.18	0.140
	Age ≥45 years (n=69)	54 (78.3%)	15 (21.7%)		
By preoperative VAS arm score	VAS ≤ 7 (n = 62)	53 (85.5%)	9 (14.5%)	χ² = 0.68	0.409
	VAS > 7 (n = 83)	67 (80.7%)	16 (19.3%)		
By disc herniation location	Paracentral (n = 88)	81 (92.0%)	7 (8.0%)	χ² = 4.38	0.042*
	Foraminal (n = 57)	46 (80.7%)	11 (19.3%)		

## Discussion

The present study evaluated the clinical and functional outcomes of posterior endoscopic decompression in patients with symptomatic cervical disc herniation and demonstrated significant improvements in pain relief, functional recovery, and overall patient satisfaction during the follow-up. The findings of this study support the growing evidence that posterior endoscopic decompression is an effective and minimally invasive alternative for selected patients with cervical radiculopathy caused by lateral or foraminal disc herniation.

A significant reduction in arm and neck pain was observed postoperatively, along with a marked improvement in NDI scores. The mean VAS score decreased from 7.6 preoperatively to 1.9 at 12 months, whereas the NDI score improved from 54.3 to 19.8. These findings are consistent with those of previous studies by Ruetten et al. [13] and Liawrungrueang et al. [14], who reported substantial postoperative pain reduction and functional improvement after posterior endoscopic cervical decompression. The observed clinical improvement may be attributed to direct decompression of the affected nerve root, reduced soft tissue disruption, and preservation of cervical spinal stability associated with the posterior endoscopic approach.

The overall success rate based on the Modified MacNab criteria in the present study was 82.8%, with excellent or good outcomes reported in the majority of patients. Similar success rates have been reported in the literature. A systematic review and meta-analysis by Zhang et al. [9] demonstrated favorable clinical outcomes and high patient satisfaction rates following percutaneous endoscopic cervical discectomy. The minimally invasive nature of endoscopic surgery contributes to reduced postoperative pain, shorter hospitalization, smaller incisions, and faster rehabilitation, which may collectively improve the patient-reported outcomes.

The majority of disc herniations in the present study occurred at the C5-C6 and C6-C7 levels, which is in agreement with the known biomechanical susceptibility of the lower cervical spine to degenerative changes and repetitive mechanical stress [15]. Furthermore, paracentral disc herniation demonstrated significantly better outcomes than foraminal disc herniation. This finding may be attributed to the relatively more favorable anatomical access provided by the posterior endoscopic approach in paracentral lesions, allowing more direct visualization and effective decompression of the affected nerve root. In contrast, foraminal disc herniations are often associated with a narrower surgical corridor, limited working space, and closer proximity to neural structures, which can make complete decompression technically more challenging. Additionally, chronic foraminal stenosis and bony encroachment frequently coexist with foraminal disc herniations, potentially contributing to persistent symptoms and less favorable postoperative recovery. These findings highlight the importance of careful patient selection and preoperative radiological assessment when considering posterior endoscopic decompression for different cervical disc herniation subtypes. Similar observations were reported by Ahn et al. [16], who emphasized the importance of careful patient selection and surgical expertise in achieving optimal outcomes with endoscopic cervical procedures.

Although patients with a symptom duration ≤6 months demonstrated higher success rates than those with a longer symptom duration, the difference did not reach statistical significance. Nevertheless, prolonged neural compression may contribute to chronic inflammatory changes, nerve root fibrosis, and delayed neurological recovery, potentially affecting surgical outcomes [17]. Therefore, early surgical intervention following failed conservative therapy may result in better symptomatic improvement and functional recovery.

This minimally invasive posterior endoscopic approach offers several biomechanical advantages over anterior cervical discectomy and fusion. The preservation of motion segments and avoidance of fusion-related complications such as adjacent segment degeneration, implant failure, and dysphagia represent important benefits of this technique. In addition, reduced muscle dissection and minimal osseous removal help preserve spinal alignment and postoperative cervical mobility [18]. These advantages make posterior endoscopic decompression particularly suitable for younger and more active patients with unilateral radiculopathy with preserved cervical stability.

Despite these favorable findings, this procedure remains technically demanding and has a significant learning curve. Adequate training, meticulous surgical planning, and proper patient selection are essential for minimizing complications and ensuring satisfactory outcomes. The use of advanced endoscopic visualization systems and continuous irrigation also contribute to improved intraoperative visualization and reduced tissue trauma.

This study has several clinical implications. Posterior endoscopic decompression may be considered an effective and less invasive surgical alternative for appropriately selected patients with cervical disc herniation, particularly those with unilateral foraminal or paracentral pathology refractory to conservative treatment. The procedure offers significant pain relief, improved functional outcomes, and high patient satisfaction while preserving cervical motion and minimizing surgical morbidity.

However, this study has several limitations. First, it was conducted at a single center without a comparative control group, which limits the ability to directly compare posterior endoscopic decompression with other established surgical techniques and may reduce the generalizability of the findings. Second, the follow-up period was limited to 12 months. Although sufficient for evaluating short-term clinical and functional outcomes, a longer follow-up period of 2-5 years would be necessary to adequately assess long-term recurrence, adjacent-segment degeneration, and the durability of clinical improvement. Third, routine postoperative MRI or CT evaluation was not performed in all patients; therefore, surgical success was assessed primarily using validated clinical and functional outcome measures rather than systematic radiological confirmation of decompression. Additionally, the study did not systematically evaluate factors such as surgeon experience, learning curve effects, operative duration, perioperative management, or postoperative rehabilitation protocols, all of which may influence outcomes and affect reproducibility across different institutions. Future multicenter controlled studies with longer follow-up periods, standardized perioperative protocols, and comprehensive radiological assessment are warranted to further validate the long-term efficacy, safety, and reproducibility of posterior endoscopic decompression.

## Conclusions

Within the limitations of this single-center observational study, posterior endoscopic decompression was associated with significant improvements in pain, functional recovery, and patient-reported outcomes in patients with symptomatic cervical disc herniation. Favorable outcomes were observed in the majority of appropriately selected patients, with paracentral disc herniation demonstrating better outcomes than foraminal disc herniation. However, the absence of a control group, lack of randomization, and limited follow-up duration preclude conclusions regarding comparative effectiveness or superiority over other surgical techniques. Further multicenter controlled studies with longer follow-up are needed to confirm the long-term efficacy, safety, and reproducibility of this approach.
